# Optimization Method of Floating Fixture Layout for Distortion Control of Low-Stiffness Thin-Walled Beams

**DOI:** 10.3390/ma17174226

**Published:** 2024-08-27

**Authors:** Junping Feng, Jiawei Wang, Zhuang Mu, Yifei Gu, Zongyang Du, Wenbo He, Kean Aw, Yinfei Yang

**Affiliations:** 1 School of Mechanical Engineering, Jiangsu University of Technology, Changzhou 213001, China; jxfjp@jstu.edu.cn (J.F.); 15651318505@163.com (J.W.); guyifei2022@163.com (Y.G.); 18238852118@163.com (Z.D.); 2 College of Mechanical and Electrical Engineering, Nanjing University of Aeronautics & Astronautics, Nanjing 210016, China; 18896790207@163.com; 3 Nanjing Hangdian Intelligent Manufacturing Technology Co., Ltd., Nanjing 210014, China; Wenbo_He@outlook.com; 4 Yangtze River Delta Intelligent Manufacturing Innovation Center, Nanjing 210014, China; 5 Department of Mechanical and Mechatronics Engineering, University of Auckland, Auckland 1010, New Zealand; k.aw@auckland.ac.nz

**Keywords:** low-stiffness thin-walled beams, floating fixture, layout optimization, elastic deformation control

## Abstract

The aim is to reduce the elastic deformation of the web and side walls of low-stiffness thin-walled beams when the floating fixture method is used. This paper takes the number and position of fixture points as the optimization variables, establishes a calculation model of elastic deformation, and constructs the objective function of maximum total elastic deformation. An optimized solution utilizing the augmented multiplier method is employed, which forms the basis for the fixture layout optimization method to reduce the elastic deformation of low-stiffness thin-walled beams. A theoretical calculation, simulation analysis, and the fixture layout optimization of total maximum elastic deformation were completed using an aluminum alloy low-stiffness thin-walled beam as an example. The results show that based on the optimized layout, the average relative error between the calculated value and the simulated value of total maximum elastic deformation is 17.43%, and the simulated value of maximum elastic deformation is reduced by 48.49% after optimizing the fixture layout. The measured value is reduced by 0.02 mm on average, and deformation is reduced by 74.07%, which verifies the effectiveness of the floating fixture layout optimization control of machining elastic deformation proposed in this paper.

## 1. Introduction

The extensive use of sizeable thin-walled beam structural parts has been shown to reduce the number of aircraft components and improve production and assembly efficiency [1,2]. However, after undergoing a series of processes, a blank thin-walled beam retains a significant amount of initial residual stress [3,4,5]. This can lead to elastic deformation during the cutting process, becoming a critical challenge that needs to be addressed [6,7]. Therefore, finding solutions to control this elastic deformation is crucial at present.

The continuous improvement of fixture technology is conducive to solving the problem [8,9,10]. The design and optimization of fixture layouts oriented toward elastic deformation control have been studied many scholars in China and abroad. Michael et al. [11] used parameterized finite element models and a hybrid discrete–integer genetic algorithm to optimize the layout of floating fixtures to improve the quality of workpieces; Fountas et al. [12] conducted research on globally optimal tool paths for complex surfaces, focusing on addressing the issues of machining errors and cutting angle smoothness; Park et al. [13] used virtual machining technology to analyze the distortion of thin-walled aluminum alloy parts, resulting in shorter development cycles, while ensuring the conditions of machining distortion; Hajimiri et al. [14] combined genetic algorithms with finite element simulation analysis to optimize the layout and fixture sequence of thin-walled workpieces fixtures to reduce the maximum elastic distortion of the workpieces under the given forces of fixturing and cutting. Errors at the assembly stage generate malfunctions in the operation of components. The dynamic characteristics are particularly sensitive to this. According to Karpenko et al. [15], to improve the adaptive properties of a system, the dynamic characteristics of the material should be tested using frequency analysis.

Liu et al. [16] developed floating fixtures for cutting aircraft stringers by adjusting the positions of the pressure plates and the support blocks with motors to accommodate different-sized stringer workpieces; Wang et al. [17] studied the influence of fixture suction force on fixture distortion and used finite element analysis to study the distortion of a jet bridge under only its own gravity and suction-cup suction force. They aimed to minimize the distortion of the jet bridge during the assembly process, while meeting the requirements of aircraft assembly errors. Yue et al. [18] used an established machining state monitoring model to accurately monitor the cutting state of aerospace thin-walled parts to control the machining distortion of thin-walled parts; Men et al. [19] used a BP neural network prediction model with positioning element positions as decision variables and applied genetic algorithms to optimize the positioning layout to minimize the total strain energy and achieve the aim of controlling the machining distortion of thin-walled parts.

In order to improve the machining accuracy of a workpiece, the optimization of positioning and fixture elements; the optimization of tool paths; the adoption of virtual machining technology; layout and fixture sequence optimization; the development of floating fixtures; the analysis of fixture forces; the monitoring of the machining status; and the minimization of overall strain energy have all been widely studied and have achieved good results. However, the machining process of a workpiece represents a real-time change in a system, and using floating fixture technology can be an excellent solution to the dynamic release of strain energy inside the workpiece during machining [20,21]. On this basis, it is proposed to reduce the elastic deformation of low-stiffness thin-walled beams as the goal, establish a mathematical model with the number and location of fixture points as the design variables, and construct the objective function of maximum total elastic deformation. The objective function is optimized using the augmented multiplier method [22,23], and the design variables that satisfy the constraints are solved. The theoretical calculation and finite element simulation of the maximum elastic deformation of an aluminum alloy low-stiffness thin-walled beam fixture with an optimized layout are carried out to verify the accuracy of this paper’s elastic deformation calculation model. Finally, a fixture layout optimization test is carried out according to the existing conditions to verify the effectiveness of the method proposed in this paper.

## 2. Optimization of Fixture Layout

### 2.1. Construction of an Objective Function

Most of the sizeable thin-walled beam parts have a cavity structure. Such structural parts are usually large, with more cavities and a high material removal rate, resulting in poor machining rigidity and quick deformation during cutting [24,25,26]. The optimization of the fixture layout is conducive to reducing the elastic deformation of thin-walled beams with low stiffness. For the accurate positioning of the machined parts and the reasonable arrangement of the fixture layout, the constraint analysis of the part fixture system is required, including balance constraint, stability constraint, and constraint on the direction and the magnitude of the fixture force [10,27].

**Figure 1:**
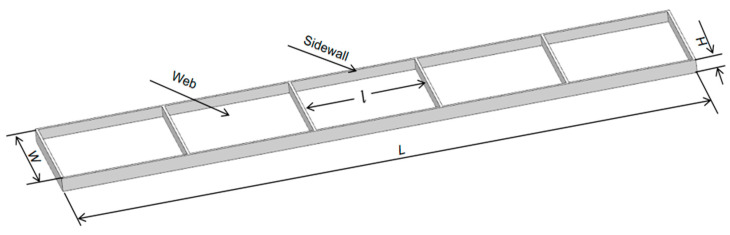
Thin-walled beams of groove structure.

To ensure that the workpiece meets the machining accuracy requirements, it is necessary to improve the floating fixture module to improve the rigidity of the thin-walled structure so that elastic deformation is controlled within the allowable range. As shown in Figure 1, a typical slot cavity structure is composed of a groove web and a side wall connection, so the maximum deformation of the center rectangular plate and the maximum deformation of the side wall structure can be approximated by a specific method of superposition using a calculation to obtain the maximum elastic deformation of the workpiece under the action of a cutting force.

The total elastic deformation of beam structural parts can be seen as the combination of both the web surface and side wall deformation. This paper uses a structural analysis approach to break down the stiffness calculation of beam structures to solve the deflection of thin plate, fixed beam, and cantilever beam models. The optimization of the fixture layout is then carried out by considering the maximum total elastic deformation of the structure as the objective function.

(1) Calculation of thin plate model

**Figure 2:**
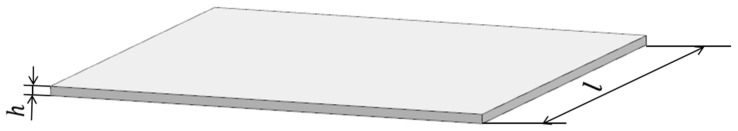
Thin plate.

Usually, the plane equidistant from the upper surface and the lower surface of the plate is called the middle surface of the plate, and the minimum side length of the middle surface is *l*. If the thickness of the plate is *h*, when *h*/*l* is less than 1/5, it can be calculated according to the thin-plate theory. According to the above theory, the web of the beam workpiece can be regarded as a thin plate, and the deformation of the web structure can be calculated and solved by using the theory of the small deflection deformation of a thin plate, as shown in Figure 2.

For rectangular thin plates, according to the equilibrium condition of the plate element and the relationship of elastic deformation, the governing equation is as follows:

(1) $$\frac{\partial^{4}\omega }{\partial x^{4}}+2\frac{\partial^{4}\omega }{\partial x^{2}\partial y^{2}}+\frac{\partial^{4}\omega }{\partial y^{4}}=\frac{q\left(x,y\right)}{D}$$

(2) $$D=\frac{Eh^{3}}{12\left(1-v^{2}\right)}$$

where $\omega \left(x,y\right)$ is the mid-surface deflection, *q* (*x*, *y*) is the unit area load, *D* is the bending stiffness of the plate, *v* is the Poisson’s ratio of the workpiece material, *h* is the thickness of the plate, and E is Young’s modulus.

**Figure 3:**
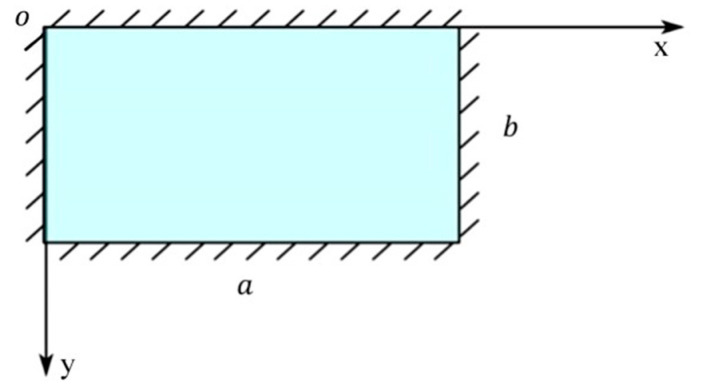
Perimeter simply supported rectangular thin plate.

The web region enclosed by the four fixture points is attached to the more stiffened side walls, so deformation is calculated according to the perimeter-supported beam rectangle, as shown in Figure 3.

The Navier solution of the perimeter simply supported rectangular plate is used, assuming the following solution of Equation (1):

(3) $$\omega =\sum_{m=1}^{\infty }\sum_{n=1}^{\infty }a_{mn}\sin\frac{m\pi x}{a}\sin\frac{n\pi y}{b}$$

where *m* and *n* are any integers, *a* is the long side of the rectangular board, and *b* is the short side. To determine the coefficient *a_mn_*, the load *q* (*x*, *y*) per unit area can be expanded as follows:

(4) $$q\left(x,y\right)=\sum_{m=1}^{\infty }\sum_{n=1}^{\infty }A_{mn}\sin\frac{m\pi x}{a}\sin\frac{n\pi y}{b}$$

where

(5) $$A_{mn}=\frac{4}{ab}\int_{0}^{a}\int_{0}^{b}q\left(x,y\right)\sin\frac{m\pi x}{a}\sin\frac{n\pi y}{b}dxdy$$

By substituting (3) and (4) into Equation (1), and then comparing the corresponding coefficients on both sides of the equation, we obtained the following general solution of the peripheral simply supported rectangular thin plate flexural equation:

(6) $$\omega =\frac{1}{\pi^{4}D}\sum_{m=1}^{\infty }\sum_{n=1}^{\infty }\frac{A_{mn}}{{\left(\frac{m^{2}}{a^{2}}+\frac{n^{2}}{b^{2}}\right)}^{2}}\sin\frac{m\pi x}{a}\sin\frac{n\pi y}{b}$$

For the concentrated force *P* acting on the point (ξ,η) on the plate plane, there is

(7) $$A_{mn}=\frac{4P}{ab}\sin\frac{m\pi \xi }{a}\sin\frac{n\pi \eta }{b}$$

Substituting (7) into (6) yields the following solution for the deflection of the point (ξ,η) on the plane of the peripherally simply supported rectangular thin plate under the action of a concentrated force *P*:

(8) $$\omega =\frac{4P}{\pi^{4}abD}\sum_{m=1}^{\infty }\sum_{n=1}^{\infty }\frac{\sin\frac{m\pi \xi }{a}\sin\frac{n\pi \eta }{b}}{{\left(\frac{m^{2}}{a^{2}}+\frac{n^{2}}{b^{2}}\right)}^{2}}\sin\frac{m\pi x}{a}\sin\frac{n\pi y}{b}$$

According to Equation (8), it can be seen that for a peripherally supported rectangular thin plate under the action of a concentrated force *P*, the point of maximum deflection is at the center of the plate, and, in general, to simplify the calculation, its maximum deflection can be treated as follows:

(9) $$\omega_{1}=K_{\omega }\frac{Pb^{2}}{Eh^{3}}$$

For Poisson’s ratio *v* = 0.3, the values of the coefficient $K_{\omega }$ as a function of the sheet aspect ratio *a*/*b* are shown in Table 1.

When changing the workpiece material, the Poisson’s ratio of the material will change. If the Poisson’s ratio *v*′ of the new material is used and the deflection is $\omega^{\prime }$, the following conversion formula can be used to calculate its maximum deflection:

(10) $$\omega_{1}^{\prime }=\frac{1-v^{\prime 2}}{1-v^{2}}\omega_{1}$$

(2) Calculation of the fixed beam model

**Figure 4:**
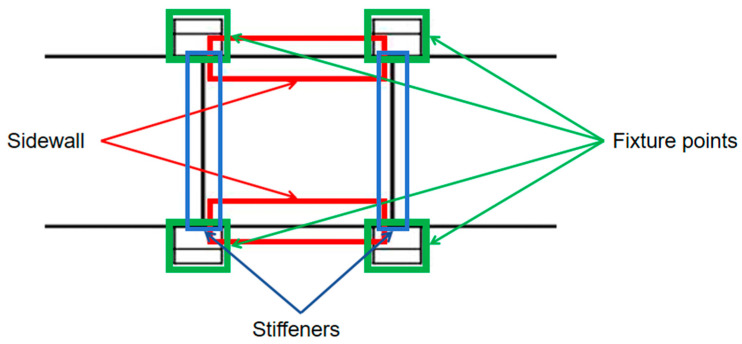
A model of the fixed beams at both ends of the side wall next to the web plate.

Since the deformation of the side wall area surrounded by four fixture points can be calculated by the deflection formula of the beam structure, and floating fixture modules clamp the two ends of the side wall, the structural model of the beam with two ends fixed can be adopted, as shown in Figure 4. The flexural line is shown in Equation (11).

(11) $$\omega =\frac{Px}{192EI}\left(3l^{2}-4x^{2}\right)$$

From Equation (11), the deflection is highest at the location of the midpoint of the beam, i.e., when *x*, there is a maximum deflection of

(12) $$\omega_{2}=\frac{Pl^{3}}{192EI}$$

where *I* is the moment of inertia of the cross-section of the beam structure, *l* is the span of the beam, and in this model, *l* is the distance between the two floating fixture modules.

(3) Calculation of cantilever beam model

**Figure 5:**
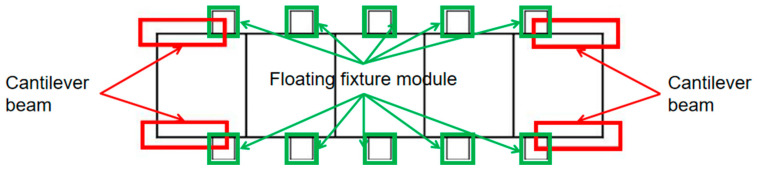
Cantilever beam model for boundary side walls.

The two ends of the long beam part can be represented as a cantilever beam model, as shown in Figure 5, and its maximum deflection will appear at the outermost end away from the floating fixture module. Maximum deflection is calculated in Equation (13).

(13) $$\omega_{3}=\frac{Pl^{3}}{3EI}$$

**Figure 6:**
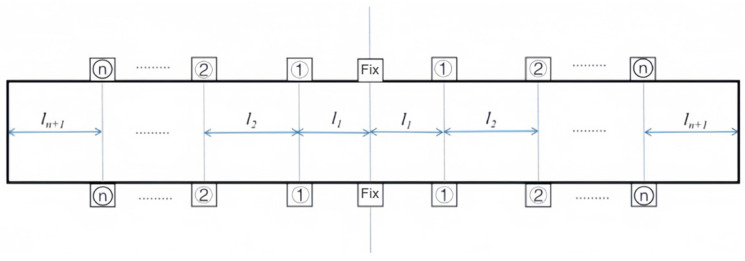
Schematic diagram of fixture layout model.

Web deformation $\omega_{1}$, side wall deformation $\omega_{2}$, and outer end cantilever beam deformation $\omega_{3}$ are superimposed to obtain the total maximum elastic deformation $\omega_{z}$ of the beam structural member, as calculated by the formula shown in (14). Let the overall length of the beam be *L*; there are *n* pairs of floating fixture modules distributed along the center line left and right, and the distance between each adjacent two pairs of floating fixture modules is *l*_1_, *l*_2_…*l_n_*_+1_. The model is shown in Figure 6.

(14) $$\omega_{z}=2[\sum_{i=1}^{n}(K_{\omega }\frac{Pl_{i}^{2}}{Eh^{3}}+\frac{Pl_{i}^{3}}{192EI})+\frac{Pl_{n+1}^{3}}{3EI}]$$

Equation (14) is the objective function to be optimized; when the number of floating fixture modules is determined, the corresponding mechanical optimization design algorithms can be used to solve Equation (14) for the extreme value to complete the optimization of the floating fixture layout.

### 2.2. Fixture Layout Optimization Method

**Figure 7:**
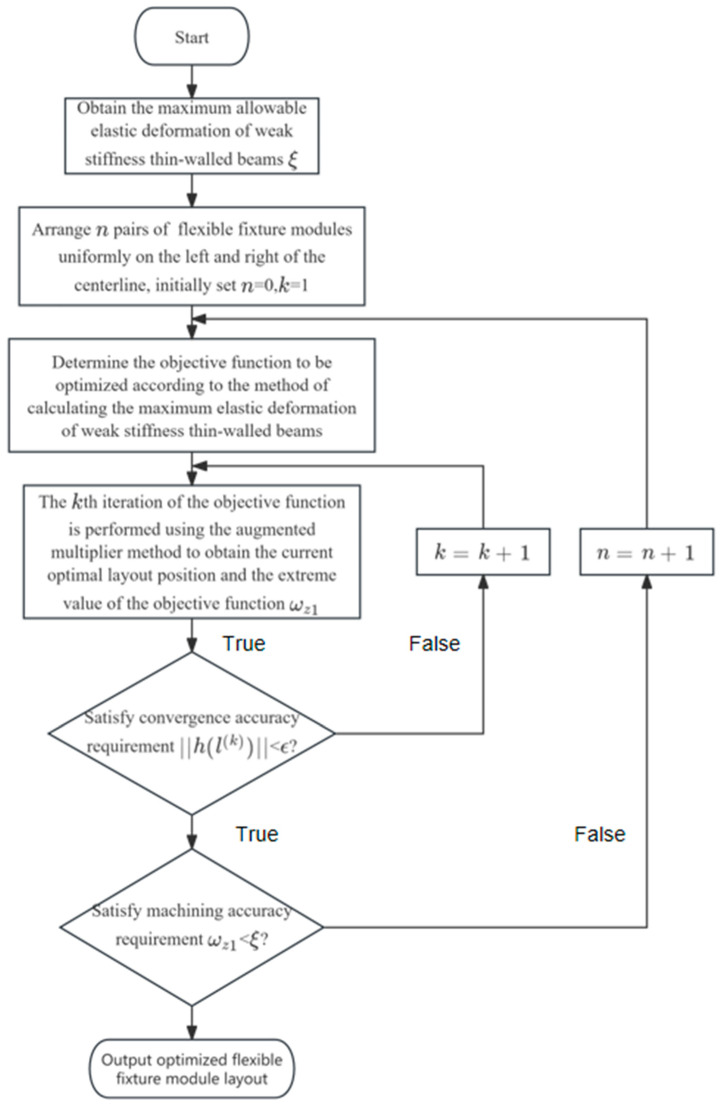
Fixture layout optimization flowchart.

Typical low-stiffness thin-walled beams are prone to elastic deformation when milling. In this section, based on positioning determination, the optimization of the fixture layout is carried out from the perspective of reducing the elastic deformation generated by the machining of thin-walled beam components. The optimization strategy of the fixture layout of beam thin-walled components is given, as shown in Figure 7. When initially developing the fixture program, the left and right distributions of *n* pairs of floating fixture modules were initially set as *n* = 0. We adopted the augmented multiplier method of optimization of the objective function (14) to obtain the optimal layout of the floating fixture module at this point and the extreme value of the objective function $\omega_{z1}$. We assessed the extreme value of the total maximum elastic deformation $\omega_{z1}$ and checked if it was less than or equal to the maximum permissible elastic deformation of part ξ, and if was in accordance, it immediately output the number of floating fixture modules and the coordinates of the optimal layout position; if not, the number of floating fixture modules steadily increased, i.e., *n* = *n* + 1. The above steps were repeated until the typical low-stiffness thin-walled beam achieved machining accuracy.

### 2.3. Augmented Multiplier Method

Presently, the augmented multiplier method is one of the mainstream algorithms used to solve the constrained extreme value problem. The augmented multiplier method is used to optimize the fixture layout of the floating fixture method and determine the optimal number and placement of floating fixture modules. The objective function of the original system is as follows:

(15) $$m\;in\;\omega_{z}\left(l_{1},\;l_{2}\ldots l_{n+1}\right)$$

(16) $$subject\;to\;h\left(l\right):\sum_{i=1}^{n+1}l_{i}-\frac{L}{2}=0$$

By introducing the penalty factor *r* and the Lagrange multiplier λ, the augmented multiplier function of the equation-constrained optimization problem is constructed as follows:

(17) $$M\left(l,\lambda ,r\right)=\omega_{z}\left(l\right)+\frac{r}{2}\sum_{p=1}^{m}{\left[h_{p}\left(l\right)\right]}^{2}+\sum_{p=1}^{m}\lambda_{p}h_{p}\left(l\right)\;\left(m=l\right)$$

Therefore, the objective function of the new system for the optimization of the fixture layout is

(18) $$minM\left(l,\lambda ,r\right)=\omega_{z}\left(l_{1},\;l_{2}\ldots l_{n+1}\right)+\frac{r}{2}{\left(\sum_{i=1}^{n+1}l_{i}-\frac{L}{2}\right)}^{2}+\lambda \left(\sum_{i=1}^{n+1}l_{i}-\frac{L}{2}\right)$$

The multiplicative iterative correction formula is

(19) $$\;\lambda^{\left(k+1\right)}=\lambda^{\left(k\right)}+rh\left(l^{\left(k\right)}\right)$$

The increasing formula of the penalty factor is

(20) $$r^{\left(k+1\right)}=\beta r^{\left(k\right)}$$

In the iterative process of optimization calculation, the penalty factor *r* does not need to tend to infinity. When *r* increases to a certain value, λ tends to $\lambda^{*}$, and thus the extreme point of the augmented multiplier function also approaches the optimal solution of the optimization problem of the fixture layout. Mathematical model optimization based on the augmented multiplier method is divided into the following seven steps.

Step 1: Select the appropriate design variable initial value *l*^(0)^, penalty factor *r*^(0)^, and convergence accuracy ε; let $\lambda^{\left(0\right)}$ = 0 and the number of iterations *k* = 0.

Step 2: According to the original objective function and constraints, construct a new objective function M(l,λ,r).

Step 3: Optimize the iterative calculation of the objective function, calculate minM(l,λ,r), and obtain feasible values for $l_{1}^{\left(k\right)}$, $l_{2}^{\left(k\right)}\ldots l_{n+1}^{\left(k\right)}$, $\lambda^{\left(k\right)}$ and the objective function value $\omega_{z1}$ in each iteration.

Step 4: According to Equation (19), multiplier iteration correction is performed to calculate $\lambda^{\left(k+1\right)}$.

Step 5: Calculate $\left||h(l^{\left(k\right)})|\right|$ to determine whether the convergence accuracy ε is satisfied. If it is, begin Step 7; otherwise, begin Step 6.

Step 6: Calculate the penalty factor iteratively according to Equation (20); let *k* = *k* + 1, and then begin Step 3.

Step 7: Output the current extreme point combination of $l_{1}^{*}$, $l_{2}^{*}\ldots l_{n+1}^{*}$ and $\omega_{z}$ function values.

Through the above seven steps, the floating fixture layout, which is conducive to reducing the elastic deformation of low-stiffness thin-walled beams, can be obtained.

## 3. Simulation of Example

**Figure 8:**
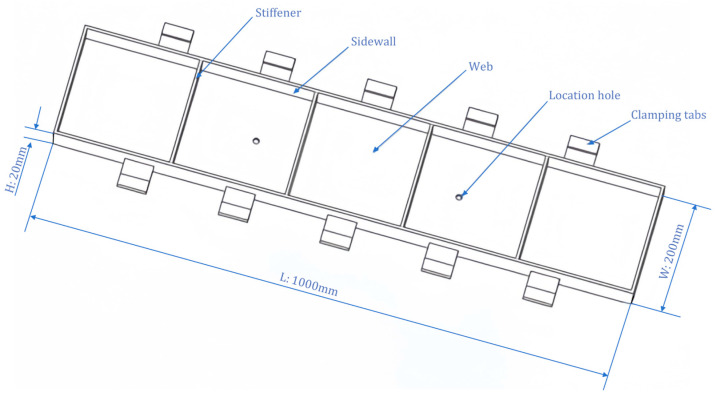
An aluminum alloy low-stiffness thin-walled beam.

In this section, an aluminum alloy low-stiffness thin-walled beam is taken as the research object to obtain the number and position of fixture points. The structure and specific dimensions of the low stiffened thin-walled beam are shown in Figure 8, with dimensions of 1000 mm × 200 mm × 20 mm, a slot depth of 16 mm, a side wall and web thickness of 4 mm, and a symmetrical structure. According to the structural characteristics of this beam part, they can be symmetrically set along the two long edges. Adopting the “one side, two pins” positioning method in the six-point positioning principle, the two zero-point positioning modules should be arranged close together in the main deformation direction of the beam parts near the two indicated holes.

The initial fixture point layout is set up with *n* pairs of floating fixture modules uniformly distributed along the left and right sides of the center line, and *n* = 0 in the initial state. According to the method of calculating the elastic deformation of low-stiffness thin-walled beams under a cutting force in Section 2.1 and the optimization method of the number of fixture points based on the incremental and generalized multiplier method described in Section 2.3, *n* is iteratively increased one by one to obtain the extreme value of total elastic deformation using the logarithm of different fixture modules.

**Figure 9:**
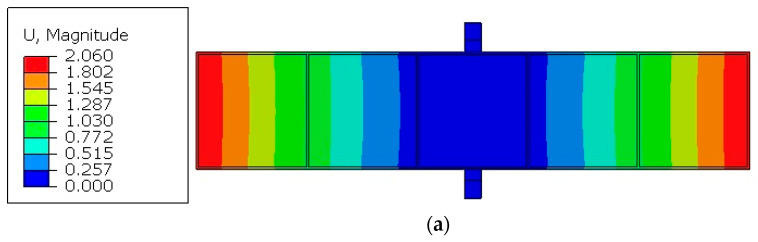

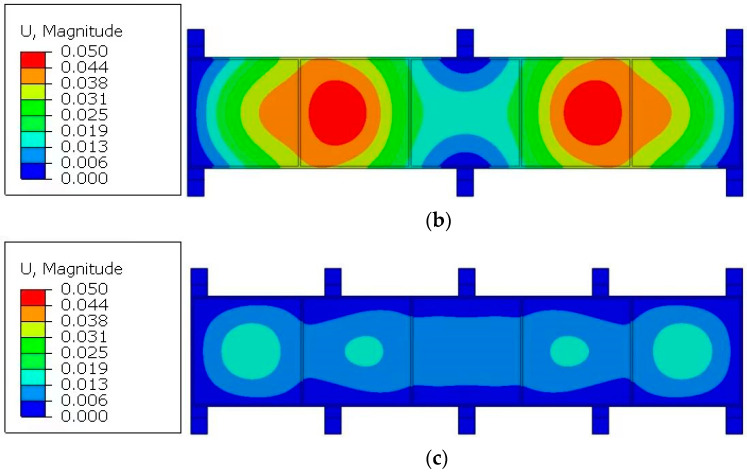
The simulation results of the total elastic deformation of thin-walled beams optimized using different logarithms of the floating fixture module. (**a**) The simulation result of total elastic deformation when *n* = 0 (unit: mm). (**b**) The simulation result of total elastic deformation when *n* = 1 (unit: mm). (**c**) The simulation result of total elastic deformation when *n* = 2 (unit: mm).

To improve the reliability of the layout optimization results, this paper then uses finite element software(Abaqus 2021) to carry out an example simulation to obtain the extreme value of total maximum elastic deformation corresponding to the logarithm of the fixture module. Through the output results and the example simulation results, it can be seen that when *n* = 2, the value of maximum elastic deformation at this time meets the requirements of machining accuracy and elastic deformation control within 0.05 mm. We output the results of the fixture layout at this time; the position coordinates of the tabs are *x*_1_ = 29.3 mm, *x*_2_ = 263.9 mm, *x*_3_ = 500.0 mm, *x*_4_ = 736.1 mm, and *x*_5_ = 970.7 mm. The total elastic deformation of the optimized thin-walled beams using different numbers of pairs of floating fixture modules is shown in Figure 9.

Figure 9 shows the simulation results of the total elastic deformation of thin-walled beams optimized using different logarithms of the floating fixture module.

Since it is an aluminum alloy material, when using Equation (14) to calculate the total theoretical maximum elastic deformation, *P* = 300 N, and *E* = 72 GPa. *I* = *bh*^3^/12, where *b* is the width of the beam, equal to 200 mm, and *h* is the height of the beam, equal to 20 mm, and *I* = 133333.3333 mm^4^. Since the length of the selected thin-walled beam is *a* = 1000 mm, and *b* = 200 mm, from Table 1, $K_{\omega }$ = 0.0791.

**Figure 10:**
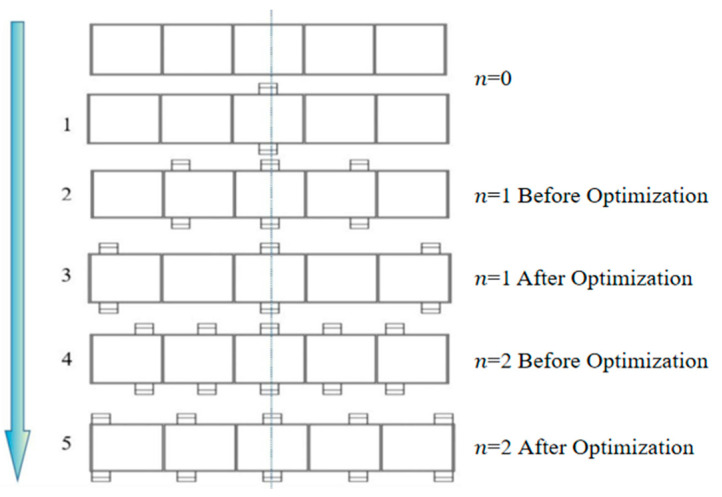
Fixture point layout optimization process.

The total maximum elastic deformation of the thin-walled beam is iteratively optimized for the calculated value of ${\;\delta }_{k}\;$ and the simulated value of ${\;\delta }_{k}^{\prime }\;$ using the optimal fixture layout, as shown in Table 2 below. The optimization process of the fixture layout is shown in Figure 10.

**Figure 11:**
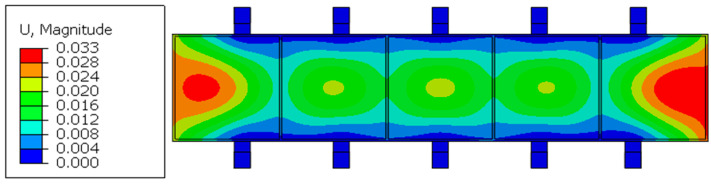
Simulation results of total elastic deformation without optimized fixture layout when *n* = 2 (unit: mm).

**Figure 12:**
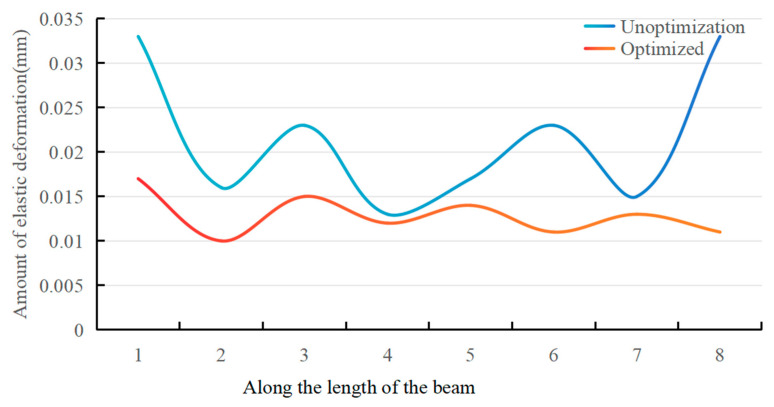
Comparison chart of deformation.

As shown in Table 2, the average relative error between the calculated value $\delta_{k}$ and the simulated value ${\;\delta }_{k}^{\prime }$ of total maximum elastic deformation is 17.43% for the different pairs of fixture points, which verifies the accuracy of the floating fixture optimization mathematical model described in Section 2.1 of this paper. When *n* = 2, the simulation results of total elastic deformation without fixture layout optimization are shown in Figure 11. Before and after the optimization of the fixture layout, the comparison of the deformation is shown in Figure 12.

As can be seen from Figure 10 and Figure 11, when *n* = 2, the maximum elastic deformation after the optimization of the fixture layout is 0.017 mm, and the maximum elastic deformation before the optimization of the fixture layout is 0.033 mm, which reflects an optimization value of 48.49%. As can be seen from Figure 12, the elastic deformation of the parts along the beam length direction with the optimized fixture layout is significantly smaller than that along the beam length direction under non-optimized conditions, and the deformation fluctuation along the beam length direction after optimization is also significantly smaller than that before optimization. This shows the effectiveness of the floating fixture optimization method for the elastic deformation control of thin-walled beams with low stiffness.

## 4. Experimental Verification

**Figure 13:**
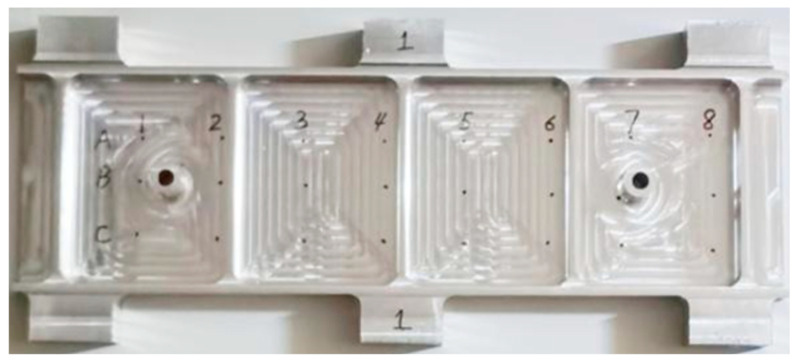
Measurement points.

**Figure 14:**
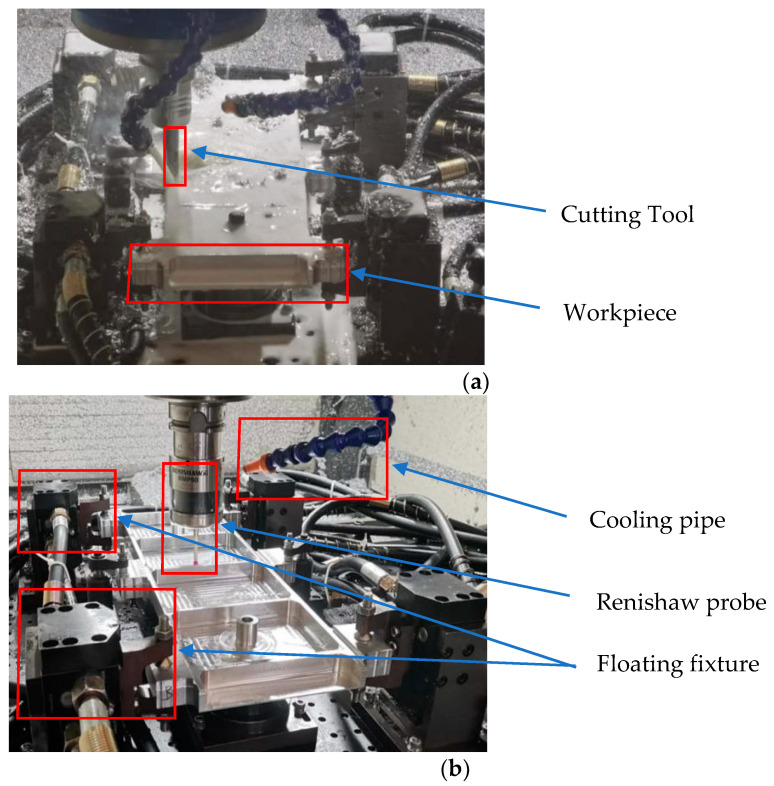
Experiment and measurement: (**a**) milling operation; (**b**) measuring distortion.

This experiment was affected by the test’s cost, the site conditions, the machine performance, and other aspects. According to the existing test conditions, the selected beam test part size is 475 mm × 140 mm × 20 mm, the side wall thickness is 5 mm, and the thickness of the web plate is 3 mm. Eight measurement points were set along the long side of the beam parts, and each column interval was 50 mm, as shown in Figure 13. This machining experiment was carried out using NingQing VC-3016G gantry CNC machining equipment (Provided by China Nanjing Ningqing CNC Machine Tool Manufacturing Co., LTD., Nanjing, China), and the tool used in the experiment was a cemented carbide φ16 end mill. A Renishaw RWP60 needle probe was used for deformation measurements. The cutting parameters are shown in Table 3, and the machining site is shown in Figure 14.

**Figure 15:**
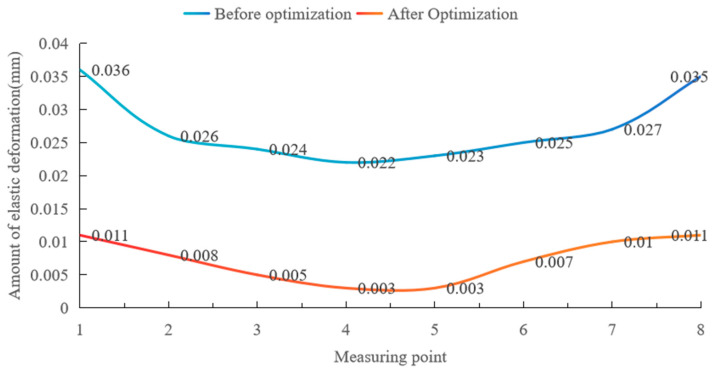
Elastic deformation at each measurement point before and after optimization of fixture layout.

This experiment measured the amount of elastic deformation at each measurement point before and after optimizing the fixture layout, as shown in Figure 15 and Table 4 below.

Due to the impact of the measurement environment and equipment accuracy, there may have been some errors in the measurement process. Therefore, the method of repeated measurement and the average value were adopted to reduce the errors as much as possible to ensure the accuracy of the data.

According to Figure 15 and Table 4, in the experiment, before the optimization of the fixture layout, the maximum elastic deformation of the thin-walled beam was 0.036 mm, and the average maximum elastic deformation was 0.027 mm. After the optimization of the fixture layout, the maximum elastic deformation of the thin-walled beam is 0.011 mm, and the average maximum elastic deformation is 0.007 mm, which is reduced by 0.020 mm, and the optimization value is 74.07%. As can be seen from Figure 15, elastic deformation along the beam length direction of the parts with the optimal fixture layout is significantly smaller than that under non-optimal conditions, and deformation along the measured parts with the optimal fixture layout is significantly smaller. It can be seen that the optimization method of the floating fixture layout for the elastic deformation control of thin-walled beams with low stiffness is practical and effective.

## 5. Conclusions

To solve the problem of the elastic deformation of a workpiece in the cutting process, this paper gives a fixture layout optimization method to reduce the elastic deformation of low-stiffness thin-walled beams. As an example, a theoretical calculation, finite element simulation, and a fixture layout optimization test are carried out on an aluminum alloy low-stiffness thin-walled beam. The main conclusions are as follows:

(1) The elastic deformation of the workpiece generated in the cutting process can be effectively reduced by adding the floating fixture module. For thin-walled beams with external dimensions of 1000 mm × 200 mm × 20 mm, a groove depth of 16 mm, and a thickness of the side walls and webs of 4 mm, it is necessary to set up two pairs of floating fixture modules symmetrically along the center of the beam.

(2) When the number of pairs of floating fixture modules is different, the relative error between the calculated value of total maximum elastic deformation $\delta_{k}$ and the simulated value $\delta_{k}^{\prime }$ is 17.43%, which verifies the accuracy of the floating fixture optimization mathematical model studied in this paper.

(3) In the fixture layout optimization test, after optimizing the fixture layout, the elastic deformation of the eight measurement points is reduced by up to 0.025 mm, with an average reduction of 0.02 mm, and deformation is reduced by 74.07%. The test effectively reduces the elastic deformation of thin-walled beams in the machining process.

## Figures and Tables

**Table 1:** The values of the coefficient $K_{\omega }$.

*a*/*b*	1	2	3	4	∞
$K_{\omega }$	0.0611	0.0788	0.0790	0.0791	0.0791

**Table 2:** Iterative results of total maximum elastic deformation for fixture layout optimization.

Logarithm of Fixture Points *n*	Fixture Module Spacing *l*/mm	Clamp Module on Edge of Beam *l_n_*_+1_/mm	$\mathrm{Calculated}\;\mathrm{Value}\;\delta_{k}$/mm	$\mathrm{Simulation}\;\mathrm{Value}\;\delta_{k}^{\prime }/mm$	Relative Error/%	Average Relative Error/%
0	0	496	2.542	2.060	18.96	
1	495	0	0.060	0.049	18.33	17.43
2	247.5	0	0.020	0.017	15.00	

**Table 3:** Cutting parameters.

Cutting Parameters	Value
Cutting depth *a_p_* (mm)	1
Cutting width *a_e_* (mm)	6
Spindle speed *n* (rpm)	800
Feed speed *f* (mm/min)	4000
Tool diameter (mm)	8
Tool tooth number (N)	3

**Table 4:** Elastic deformation reduction value at each measurement point.

	Measuring Point Number							
	1	2	3	4	5	6	7	8
Reduced value/mm	0.025	0.018	0.019	0.019	0.020	0.018	0.017	0.024

## Data Availability

The original contributions presented in the study are included in the article, further inquiries can be directed to the corresponding author.
